# Dog appeasing pheromone prevents the androgen surge and may reduce contact dominance and active submission after stressful interventions in African wild dogs (*Lycaon pictus*)

**DOI:** 10.1371/journal.pone.0212551

**Published:** 2019-03-27

**Authors:** Femke Van den Berghe, Monique C. J. Paris, Zoltan Sarnyai, Bart Vlamings, Robert P. Millar, Andre Ganswindt, Alessandro Cozzi, Patrick Pageat, Damien B. B. P. Paris

**Affiliations:** 1 Gamete and Embryology (GAME) Laboratory, College of Public Health, Medical and Veterinary Sciences, James Cook University, Townsville, QLD, Australia; 2 Institute for Breeding Rare and Endangered African Mammals (IBREAM), Edinburgh, Scotland; 3 Wageningen Livestock Research, Wageningen, The Netherlands; 4 Laboratory of Psychiatric Neuroscience, Australian Institute of Tropical Health and Medicine (AITHM) and College of Public Health, Medical and Veterinary Sciences, James Cook University, Townsville, QLD, Australia; 5 Centre for Neuroendocrinology, Department of Immunology, Faculty of Health Sciences, University of Pretoria, Pretoria, South Africa; 6 Mammal Research Institute, Faculty of Natural and Agricultural Sciences, University of Pretoria, Pretoria, South Africa; 7 Endocrine Research Laboratory, Faculty of Veterinary Science, University of Pretoria, Onderstepoort, South Africa; 8 Institut de Recherche en Sémiochimie et Ethologie Appliquée, Apt, France; 9 Centre for Tropical Environmental & Sustainability Science, James Cook University, Townsville, QLD, Australia; Centre for Cellular and Molecular Biology, INDIA

## Abstract

The endangered African wild dog (AWD; *Lycaon pictus*) is a highly social canid living in packs with a separate male and female hierarchy. Immobilisation, handling and translocations are acute stressors for AWDs, however such interventions are often needed for species management. In addition, new pack formation or temporary pack separation can lead to an increase in intra-pack aggression. The goal of this double-blinded placebo-controlled study conducted in captive zoo populations was to evaluate whether dog appeasing pheromone (DAP) reduces behavioural stress and faecal glucocorticoid metabolite levels (fGCM) normally associated with pack separation, immobilisation and reintroduction (SIR), and to assess whether this reduces aggressive behaviours and faecal androgen metabolite levels (fAM). Four packs (n = 11 males) were treated with DAP and 4 packs (n = 12 males) were treated with a placebo solution, applied at the end of anaesthesia. Behavioural interactions as well as fGCM and fAM were determined from 3 days before until 4–6 days after SIR. No effect of DAP on fGCM was observed, however, fAM increased after SIR in placebo but not DAP treated animals. Moreover, on the day of reintroduction, DAP treated packs tended to have lower rates of contact-dominance and active-submission behaviour, but higher rates of non-contact dominance behaviour. As these effects could decrease the risk of agonistic interactions, DAP may be a useful tool to help manage new pack formations and temporary pack separation.

## Introduction

The endangered African wild dog (AWD; *Lycaon pictus*) is a highly social canid living in packs of most commonly 5 to 15 adults and juveniles combined (range 2 to 27; 6.6 ± 0.8 adults) under free-roaming conditions [1]. AWDs have a complex social structure consisting of a separate male and female hierarchy, and a cooperative breeding system [1, 2]. To maintain a viable population and current genetic diversity, a meta-population management plan was introduced in South Africa in 1998, involving the reintroduction of AWDs in small protected areas and their management through regular translocations between isolated subpopulations [3]. This management plan requires frequent formation of new packs, especially since reintroductions are more successful when captive bred AWDs are released together with wild-caught animals [4, 5]. Furthermore, in the captive breeding programs of the European (EAZA) and American Association of Zoos and Aquaria (AZA), juvenile or adult animals are often translocated between zoological institutions to form new breeding pairs or packs. In addition, animals in captivity sometimes require temporary separation from their existing pack, e.g. for medical treatment. The complex social system of AWDs creates considerable challenges to this type of management. New pack formation, or disruption of hierarchical bonds and pack instability caused by temporary pack separation, can lead to aggression between animals, and have frequently resulted in serious morbidity and even mortalities [4, 6–9].

Immobilisation, handling and translocations are acute stressors for AWDs, causing increased glucocorticoid (stress hormone) output [10–13]. However, higher glucocorticoid levels have also been linked to increased aggression. In domestic dogs (*Canis familiaris*), stressed males have higher levels of dominant behaviour and aggression [14], and aggressive dogs have higher glucocorticoid concentrations than non-aggressive animals [15]. In captive AWDs, dominant males exerting active dominance, characterised by high levels of aggression, have higher glucocorticoid levels than males exerting passive dominance and low levels of aggression [10]. In free-living AWDs, dominant males engage more frequently in agonistic aggressive behaviour than subdominant animals, and have higher baseline faecal glucocorticoid metabolite (fGCM) concentrations [16]. Thus, aggression potentially seen after temporary pack separation and during new pack formation could be caused by increased glucocorticoid concentrations.

The relationship between testosterone or its precursors and aggression has been well established in a variety of species [17, 18]. For example, castration of domestic dogs often leads to a decrease in unwanted sexual behaviour, inter-male aggression, roaming, and urine marking [19]. In meerkats (*Suricata suricatta*), a social carnivore with a cooperative breeding system similar to the AWD, blockade of androgen receptors in subordinates results in a decrease in initiation of aggression and in dominant behaviour during play, but it enhances prosocial behaviour such as grooming [20]. In AWDs, dominance is often mediated by aggression. In the breeding season, alpha (and beta) males have higher androgen levels than lower ranking males [8, 16, 21], as well as higher rates of fighting [16]. Thus, a perceived stressor leading to an increase in glucocorticoid concentrations in AWDs, may cause intra-pack aggression, and is potentially associated with elevated androgen concentrations.

Appeasing pheromones could be a natural strategy to reduce stress in AWDs. Pheromones are mixtures of fatty acids that are secreted in the environment by an individual, perceived by a conspecific through inspiration into the vomeronasal organ, inducing either a physiological or behavioural response [22]. In carnivores, pheromones are secreted by a series of glands in the facial area, on the feet, in the perianal, genital and mammary region, or secreted in the urine or faeces [22]. In a wide range of mammals such as horses, cows, sheep, goats, dogs and cats, the structure and function of some pheromones have been identified and, for some, synthetic forms have been produced that prove useful to decrease stress or modify unwanted behaviour [23–25]. Mammary appeasing pheromones (nipple-finding pheromones) are known to be secreted in the above-mentioned mammals. In dogs, they are secreted by sebaceous glands in the intermammary sulcus of lactating bitches from 3–4 days post-partum until 2–5 days post-weaning; giving a calming effect on puppies [22]. The synthetic analogue of this pheromone, known as Dog Appeasing Pheromone (DAP; Adaptil, CEVA), reduces stress and unwanted behaviour (e.g. house soiling, vocalisation during isolation, or pacing) during fear-inducing situations in adult dogs [26–33] and puppies [34–37]. Further, DAP has been shown to be as effective as clomipramine, a clinically used tricyclic antidepressant, in the treatment of separation anxiety in domestic dogs [38]. Moreover, as pheromones are naturally occurring substances secreted by the target species, one of the major advantages of their use in behavioural management is the absence of toxicity or side effects [38]. However, most studies performed in domestic dogs only use behaviour to evaluate the effect of DAP. As such, more research is needed to investigate the underlying physiological response to pheromone treatment.

A preliminary study indicates that AWDs might be capable of perceiving DAP [9]. DAP infused collars tended to reduce baseline fGCM concentrations in 2/3 of treated individuals. Moreover, the use of DAP collars and sprays before new pack formation tended to result in relatively low levels of aggression [9]. However, these preliminary trials lacked appropriate control groups and had small sample sizes. Thus, further testing is needed to determine the effectiveness of DAP for the behavioural management of AWDs. The goal of this study was to characterise the effect of minor medical intervention and temporary pack separation on behaviours and hormones associated with stress and aggression, as well as determine whether DAP can mitigate these deleterious effects. We hypothesised that after separation, immobilisation and re-introduction (SIR); an intervention compilation which should be stressful to AWDs, DAP-treated animals should have (a) lower fGCM levels; (b) lower levels of intra-pack aggression-associated behaviour and increased levels of submissive and affiliative behaviour; and (c) lower faecal androgen metabolite (fAM) levels than control animals.

## Materials and methods

### Experimental design, animal capture and release

This study was approved by the James Cook University Animal Ethics Committee and by the Institutional Animal Care and Use Committees (IACUC) of the participating institutions. The study included 5 AWD packs in the USA (ABQ, Albuquerque BioPark, Albuquerque, NM; TOP, Topeka Zoo, Topeka, KS; BRK, Brookfield Zoo, Chicago, IL; BIN, Binder Park Zoo, Battle Creek, MI; and OKC, Oklahoma City Zoo, Oklahoma City, OK). Packs consisted either of 3 males (ABQ, TOP, BIN) or 3 males and 1 female (BRK, OKC). Males were scheduled for immobilisation for health assessment and sample collection [39, 40] during the 2014 pre-breeding season (4 packs; ABQ, BRK, BIN, TOP; May–early July 2014) and breeding season (ABQ, BRK, BIN, OKC; August–September 2014; Table 1). The BRK pack female was an older spayed animal euthanized due to a malignant tumour after the pre-breeding season, reducing this pack to only 3 males for breeding season evaluation, while the OKC female was pregnant at the time of sample collection. All animals had access to water *ad libitum* and were individually fed with ground horse meat, occasionally replaced by bones, whole pig or goat carcass feeding. All AWDs were housed in outdoor enclosures visible to the public (range 634–1226 m^2^) during the day with no access to off-exhibit holding areas. These holding areas were open to animals during the late afternoon, permitting free access to both areas overnight, except for the BIN pack, which was confined to their holding area (consisting of 4 separate huts each with a small outside area, connected to each other).

**Table 1:** Pack composition, treatment schedule and mean daily observation times (h) per African wild dog pack in the pre-breeding and breeding season.

		*Pre-breeding season*		*Breeding season*	
*Zoo/Pack*	*Pack composition*	*Treatment* *(4 groups/n = 12)*	*Mean (min-max) observation time (h)*	*Treatment* *(4 groups/n = 11)*	*Mean (min-max) observation time (h)*
ABQ	3 ♂	Placebo (n = 3 ♂)	2:29 (1:53–3:13)	DAP (n = 3 ♂)	3:10 (2:46–4:02)
BRK	3 ♂, 1 ♀ (in pre-breeding season)	Placebo (n = 3 ♂)	3:15 (2:35–3:55)	DAP (n = 2 ♂)	3:24 (3:09–3:34)
BIN	3 ♂	DAP (n = 3 ♂)	3:21 (2:53–3:40)	Placebo (n = 3 ♂)	2:59 (2:26–3:24)
TOP	3 ♂	DAP (n = 3 ♂)	2:02 (1:31–2:29)	-	-
OKC	3 ♂, 1 ♀	-	-	Placebo (n = 3 ♂)	4:43 (3:55–5:36)
*DAP mean daily observation time*		*2*:*59 (1*:*31–4*:*02)*			
*Placebo mean daily observation time*		*3*:*21 (1*:*53–5*:*36)*			
*Combined mean daily observation time*		*3*:*10 (1*:*31–5*:*36)*			

ABQ, Albuquerque BioPark Zoo; BRK, Brookfield Zoo; BIN, Binder Park Zoo; TOP, Topeka Zoo; OKC, Oklahoma City Zoo.

Health assessment and sample collection required the separation, immobilisation, and reintroduction after full recovery of individuals within the pack (SIR-procedure). AWDs were starved for at least 12 hours and separated into individual holding pens (with visual and olfactory contact with conspecifics) before immobilisation, which occurred over 1 or 2 consecutive days. Animals were either darted from a small distance or hand injected in a crush cage and subsequently transported to the zoo veterinary clinic for assessment. Anaesthesia was conducted according to Van den Berghe *et al*. [40]. In short, AWDs were immobilised with tiletamine/zolazepam hydrochloride (30–180 mg IM; Telazol, Zoetis Inc., MI, USA) with or without medetomidine (0–0.6 mg IM; Medetomidine HCl, ZooPharm, WY, USA), and maintained with Isoflurane (0.5–5% in 1l/min O_2_; IsoFlo, Zoetis Inc., MI, USA; Isothesia, Henry Schein Animal Health, OH, USA; or Isoflurane, MWI Animal Health, ID, USA). In cases where medetomidine was administered, AWDs were reversed with atipamezole (0.01–0.1 mg/kg, IM; Antisedan, Zoetis Inc., MI, USA) after intubation and/or at the end of anaesthesia.

After the procedure, 10 ml DAP or placebo spot-on solution (IRSEA, Apt, France) was applied to the skin and fur of each animal; 5 ml between the shoulders and 5 ml on the base of the tail. All animals immobilised from the same pack were treated with the same solution (Table 1). The placebo solution had the same composition as the DAP solution (transcutol gel), but without the pheromone. In a blinded experimental design, solutions were provided by IRSEA in 10 ml syringes marked ‘A’ or ‘B’. Details about which solution was DAP or placebo was only communicated at the end of the study, after samples were collected and analysed, data generated, and subsequent statistical analyses were completed. To control for putative seasonal effects, an equal number of packs were subjected to each treatment in both seasons (Table 1). To control for pack effects, 3 packs evaluated in both seasons were randomly assigned one treatment for each season (Table 1). In total, 4 packs (n = 11 AWD males) were treated with DAP and 4 packs (n = 12 AWD males) were treated with placebo solution (Table 1).

Animals recovered individually in their holding areas or in crates, with visual and olfactory contact with conspecifics until reintroduction. In most cases, immobilisation of the entire pack was complete within 1 day, with reintroductions performed the following morning by opening doors from individual holding areas towards the outside area, and releasing the AWDs one by one within a few seconds of each other. For the OKC pack, males were first released together, followed by the female 2 min later, once males had settled down. However, during the pre-breeding season for the BRK pack, reintroductions were performed within the holding area by opening gates between different cages. During the breeding season, the alpha male was not immobilised due to existing cardiac issues. Thus, only the 2 subdominant males were immobilised, and the alpha male was kept in the outside area during the day. All 3 males were re-introduced in their communal holding area overnight.

During the breeding season in the ABQ and BIN packs, 2 males, beta and gamma (ABQ) or alpha and beta (BIN) respectively, were sedated on day 1 and the remaining male the next day. In ABQ, the 2 subdominant males were re-introduced in the outside area on the morning the alpha male was sedated. The alpha male was isolated in his individual holding area until the following morning, with visual and olfactory contact with the 2 subdominant males that had access to the communal holding area. In BIN, all animals were isolated from each other in their holding areas over 2 days. Reintroduction was performed in the communal holding area during the evening of the second day.

### Faecal sample collection

**Fig 1:**
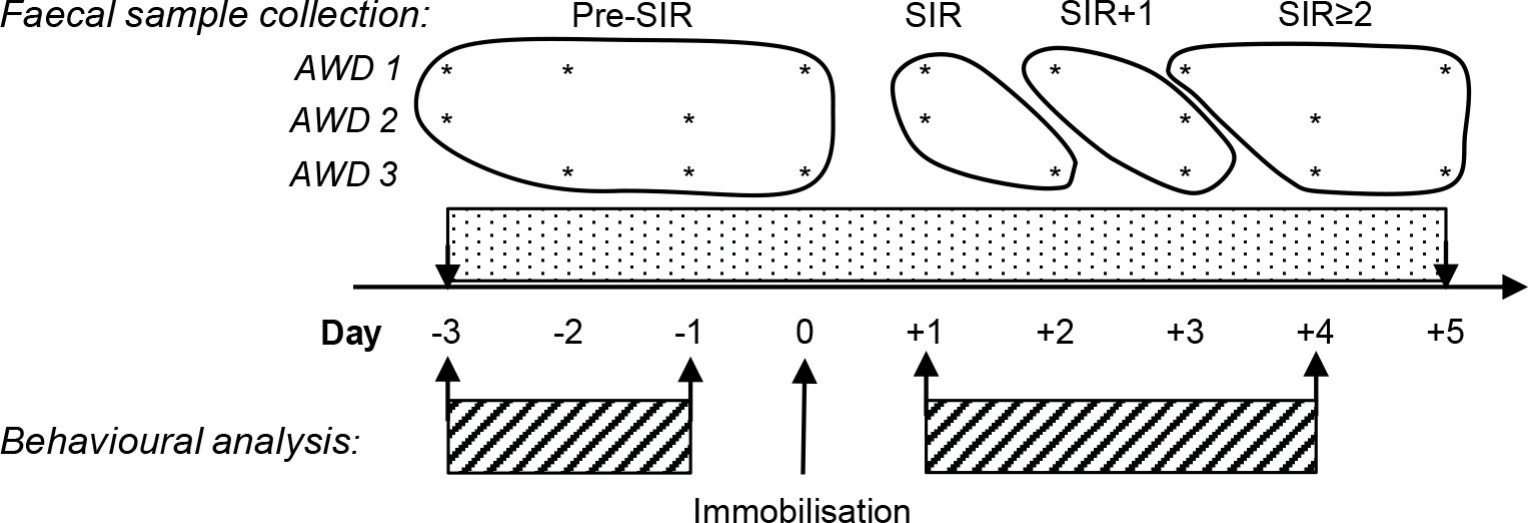
Example of faecal sample (*) and behavioural data (hatched bars) collection from each African wild dog pack. Faecal samples, collected from 3 days before until 5 days after immobilisation, were grouped as pre-SIR: samples prior to and during immobilisation; SIR: first sample after procedure; SIR+1: second sample after procedure; and SIR≥2: all subsequent samples. Behavioural observations were performed from 3 days before to 4 days after immobilisation. Day 1 includes the reintroduction of the pack.

Faecal samples were collected daily from all animals while enclosures were cleaned, from 3 days before until 5–6 days after the immobilisation of males (Fig 1). For each AWD, 3.4 ± 0.3 (mean ± SEM) faecal samples were collected over 4 days during the pre-SIR period, 1.1 ± 0.1 samples were collected over 1 day during the SIR period, 0.74 ± 0.09 samples were collected over 1 day during the SIR+1 period, and 2.4 ± 0.2 samples were collected over 3 days during the SIR≥2 period (total n = 175 samples). As enclosures were cleaned at the same time daily, all faeces were collected within 24 h and frozen immediately. Time of collection was noted. Individual-specific faeces were identified by adding non-toxic food-grade coloured plastic beads (Universal Polymers, Malaga, Australia) or glitter (local craft store, Battle Creek, MI) to an individual’s food during normal feeding times. In AWDs, gut transit time averages 22.7 ± 0.9 h [41], so faecal samples collected were assumed to indicate cumulative hormone levels from the previous day. Immediately after collection, faecal samples were placed in sealable plastic Ziploc bags and stored at -20°C until transport to Hillsdale College (MI, USA) on dry ice. Faecal samples were dried in a conventional oven (National Appliance Company, Portland, Oregon, USA) at 60°C for 21.9 ± 0.4 hours, then stored at room temperature until transport to the Endocrine Research Laboratory, University of Pretoria, South Africa, for hormone analysis.

### Faecal steroid extraction and analysis

Dried faecal samples were pulverised and put through a strainer to isolate faecal powder. Per sample, 50–60 mg of faecal powder was extracted using 3 ml 80% ethanol in water. The suspension was vortexed for 10 min and subsequently centrifuged for 10 min at 1500 *g*. The supernatant was then decanted into micro-centrifuge tubes and stored at -20°C for further analysis [42].

Faecal steroid extracts were measured for immunoreactive fGCM and fAM concentrations using an enzyme-immunoassay (EIA) technique. A biotinylated cortisol-3-CMO assay coupled with bovine serum albumin (BSA) was used for determining fGCM concentrations [43]. The sensitivity of the cortisol EIA was 1.2 ng/g dry faecal weight (DW). Intra- and inter-assay coefficients of variation of high and low concentration quality controls were 4.8% and 5.6%, and 12.2% and 13.8% respectively. To evaluate the reliability of the cortisol EIA, an ACTH stimulation test was performed during a pilot study [9]. Two female AWDs housed at the Ann van Dyk Cheetah Centre (De Wildt, South Africa) were injected with 25 IU IM synthetic ACTH (Synacthen, Novartis RSA Pty Ltd, Kempton Park, South-Africa) using a pole syringe. Faecal samples were collected from 1 day before until 5 days after ACTH administration to monitor the rise and fall in fGCM levels caused by temporal adrenal stimulation. The same samples of these 2 females were analysed for fAMs using EIA described below to exclude cross-reactivity to fGCMs. Moreover, as a biological validation, we compared the stress-associated rise in fGCMs from the first faecal sample post SIR-procedure, to the lowest fGCM values obtained pre SIR-procedure from 10 randomly selected males.

A biotinylated 3β-androstanediol (5α-androstan-3β,17β-diol) T-3-CMO assay coupled with BSA was used to determine fAM concentrations in steroid extracts [44]. The sensitivity of the testosterone EIA was 4.8 ng/g DW. Intra- and inter-assay coefficients of variation determined by measuring high and low concentration quality controls were 5.0% and 5.1%, and 8.9% and 10.9% respectively. The testosterone EIA was validated for use in the AWD by comparing fAMs in 26 samples collected from 2 females [9] with the male samples collected prior and during immobilisation (n = 100 samples). Both cortisol and testosterone assays were performed according to Ganswindt *et al*. [45].

### Effect of time and temperature on steroid metabolite concentration post-defecation

We conducted a pilot study to evaluate the effect time and temperature have on degradation of immunoreactive fAMs and fGCMs by microorganisms due to delayed sample collection and storage. Nineteen faecal samples collected from 8 males during a different study [39] were thawed, pooled (total weight 645 g), thoroughly mixed and divided into 54 equal subsamples. Half of these were held at 4°C in a refrigerator (to replicate immediate collection and prolonged storage on ice in the field during direct periods of observed defecation) and the other half were left in the sun (to replicate daily collection prior to enclosure cleaning in captivity); with fridge temperature and minimum/maximum outside temperatures monitored. Three technical replicates of each subsample were subsequently stored frozen 0 h, 1 h, 2 h, 4 h, 8 h, 16 h, 24 h, 48 h and 72 h later until processing as described above.

### Behavioural observations

**Fig 2:**
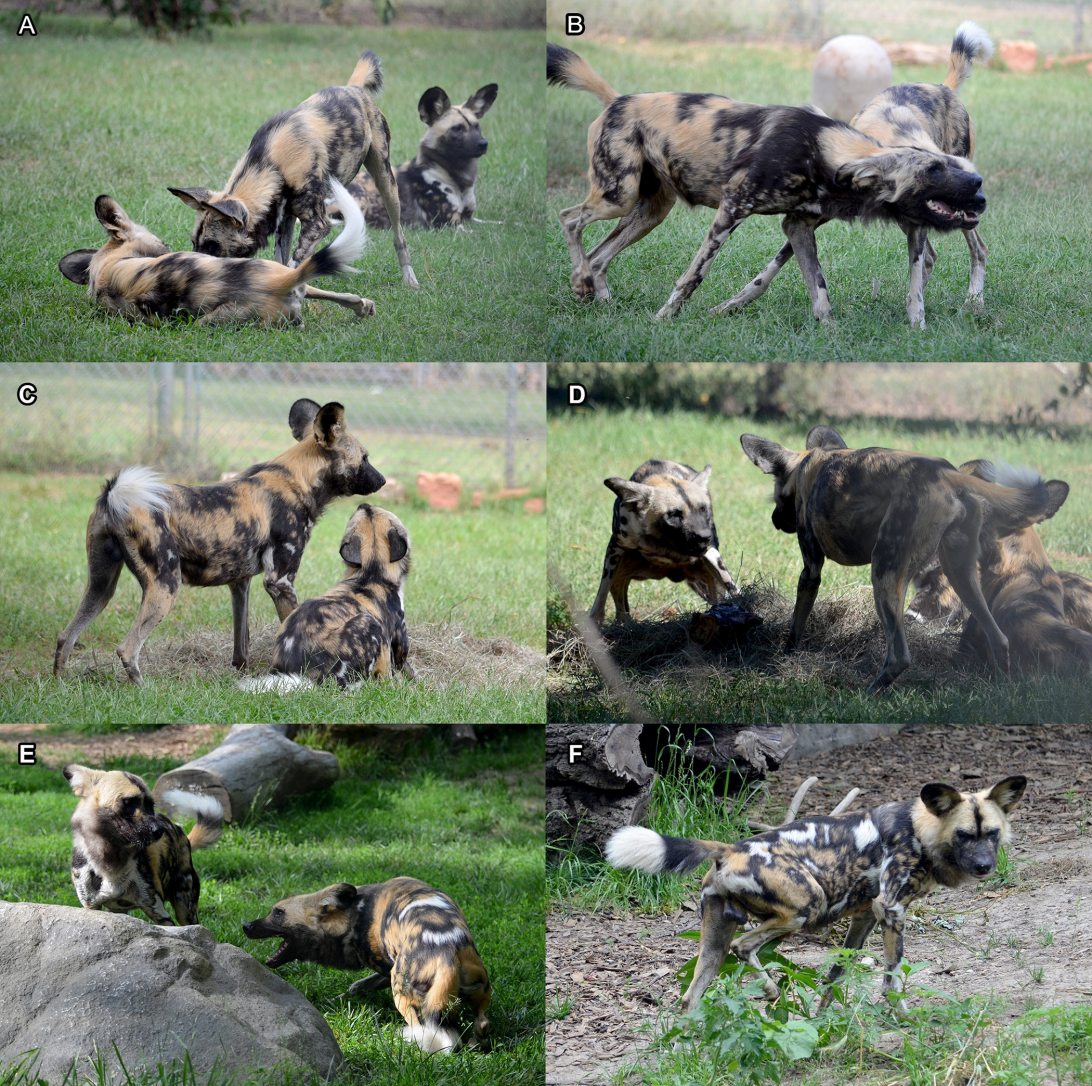
Social behaviours of captive African wild dogs (AWDs). (a) AWD approaches the scruff of a conspecific in high posture (ears forward and high tail; contact dominant behaviour), who reacts by rolling on his side (present body; passive submission). (b) 2 AWDs walk parallel touching each other’s muzzle, exhibiting the characteristic facial grin accompanied by a high-pitched giggle vocalisation (greeting; affiliative behaviour). (c) AWD on the right initiates muzzle contact with a conspecific from a low posture (low posture snout contact; active submission). (d) AWD on the right approaches a conspecific with a bone in a high posture while looking at him (food approach; non-contact dominant behaviour), who reacts by retreating in a low posture (food retreat; passive submission). (e) AWD on the right snaps towards a conspecific (snap; ritualised aggression), who reacts by retreating (shrinks back; passive submission). (f) AWD male scent marking (mark; dominant behaviour). Photographer: Femke Van den Berghe.

Behaviour was recorded using a Sony HDR-SR10 digital video camera from the public viewing area or zookeeper section from 3 days before until 4 days after immobilisation (Fig 2). Recordings started between 07:00–09:30 am (depending on zoo and day of the week), when AWDs were usually most active. The BIN and BRK packs were also observed during high activity between approximately 02:00–04:30 pm and 03:30–05:30 pm respectively. In cases were focal individuals were not visible during an observation session, the recorded time was excluded from the total daily observation time (see Table 1).

Behavioural analyses were performed blind using a modified ethogram from Vlamings ([9]; Table 2). The actor(s) and recipient(s) were classified in order to analyse social interactions between 2 or more individuals, then grouped as either severe aggression, ritualised aggression, and dominant (also including urine marking), submissive, affiliative and play behaviour (Table 2; Fig 2). Dominant interactions were subdivided into behaviours where the actor makes physical contact with the recipient (contact dominant behaviour; e.g. mount, scruff-oriented approach, or inguinal inspection), behaviours without physical contact (non-contact dominant behaviour; e.g. fixating, approach in high posture), and marking behaviour (Table 2). Submissive interactions were subdivided into behaviours where the subdominant actor initiates unprovoked submission (active submissive behaviour), and behaviour where submission is provoked by an action from a more dominant actor (passive submissive behaviour; Table 2). All social interactions in the context of food competition were excluded, as was play behaviour due to very low frequency. Hourly rates of each behaviour type were then calculated for each observation day per pack.

**Table 2:** African wild dog (*Lycaon pictus*) ethogram showing social interactions used for behavioural analysis. Modified from Vlamings (2011).

*Behaviour*	*Description*
***Severe aggressive behaviour***	
Assault*	A brusque approach at full speed, often followed by an aggressive physical contact.
Severe biting*	The actor has closed jaws and teeth having a strong hold of any part of the actor’s body e.g. legs, tail, throat or head. The bite is with full strength (uninhibited).
***Ritualised aggressive behaviour***	
Chase away	The actor walks or runs in pursuit to elicit a flee response from the recipient.
Push Down	The actor presses down the recipient by an inhibited bite in the neck.
Embrace	The actor embraces the neck of the recipient from the front, the recipient can stand on four or two hind legs.
Snout bite	The actor seizes the snout of the recipient between its jaws from the side or from above and holds it gently for a short while.
Scruff bite	Scruff orientated inhibited bite.
Snap	The actor lunges with a bite movement towards the recipient, without making contact and often with an audible sound of the jaws, when they come together.
Ritualized approach	A restrained gallop in high posture towards the recipient, not resulting in physical contact.
***Dominant behaviour***	
Aggressive vocalisation^a^	Growling.
Scruff orientated approach^b^	The actor approaches the scruff of the recipient without biting.
Stalk approach^a^	The actor slowly approaches the recipient with a prowling posture; that is with the head and neck in a straight line below the shoulder, the ears folded back, the tail relaxed or in a straight horizontal line and without losing eye-contact with the recipient.
Food approach*	The actor approaches the recipient while looking at him in the context of food acquisition.
Intervention by approach, stand or threat^a^	The actor stops an interaction between two interactants by approach, stand in between or threat towards one of the recipients respectively.
Fixating^a^	The actor looks straight at the recipient from a distance, motionless, in a high posture and with the ears forward.
Mark over urine or food	The actor secretes, with one (or both) feet lifted from the ground, a small amount of urine over a previous urine mark or food item on the ground.
Freezing^b^	The actor stands stiff with the head straight to the ground and the eyes fixated, either on the ground or on the recipient; the behaviour is shown mostly as a reaction to 'food approach'.
Inguino-genital inspection^b^	The actor initiates an inguinal contact and investigates the genitals of the recipient while the latter remains passive.
Point^a^	The actor directs, with an abrupt movement of his head or a short jump, towards the recipient.
Mount^b^	The actor places both its forepaws on the back of the recipient. It may do so from behind or from the side.
Stand over position^b^	The actor stands across a lying recipient.
Approach in high posture^a^	Moving towards the recipient in a high posture, while looking at him.
High posture snout^b^	The actor brings his nose close to or pushes it towards the nostrils of the recipient while being in a high posture.
High posture face lick^b^	The actor licks the nose, lips and mandibular region of the recipient while being in a high posture.
***Submissive behaviour***	
Escape/flight^c^	The actor runs away from the recipient, often seen during conflicts.
Retreat^c^	The actor moves away from the recipient in a low position after having been approached by him. This also includes a retreat in the context of food acquisition.
Shrink back^c^	The actor jumps back from the recipient, after being approached by him.
Avoid^c^	The actor stands aside for the recipient, after being approached by him.
Active submission^d^	A behavioural complex in which the actor actively seeks contact with a recipient by approaching him in a crouched manner with curved back and bent legs, while the tale is curled down, often wagging, and while the ears are folded back. From this position, the actor tries to contact the recipient by licking its nose.
Passive submission^c^	The actor pushes himself down in front of the recipient.
Head turning^c^	The actor turns his head and avoid eye contact with the recipient, exposing the neck region towards the recipient.
Low posture standing^c^^,^^d^	Stand in a low position, with the ears pulled back.
Approach in low posture^c^^,^^d^	The actor moves towards the recipient in a low posture while looking at him.
Low posture snout contact^c^^,^^d^	The actor brings his nose close to or pushes it towards the nostrils of the recipient while being in a low posture.
Submissive vocalisation^c^^,^^d^	Twittering, whimpering, yelping, whining vocalisations.
Present body^c^	The actor rolls on his side in front of the recipient or rolls towards him, awaiting his inspection.
Food solicit*	The actor approaches or walks in parallel with the recipient while begging for food and trying to reach for his mouth corners. There is some resemblance with 'greeting', which is an affiliative behaviour, but the context is different and the behaviour is not likely to be reciprocated.
Hoo call*	Indicative for distress.
Low posture face lick^c^^,^^d^	The actor licks the nose, lips and mandibular region of the recipient while being in a low posture.
***Affiliative behaviour***	
Close contact*	The actor stands or lies within one body length from the recipient. The recipient may be standing, sitting or lying.
Approach in neutral posture	The actor moves towards the recipient in a neutral posture while looking at him.
Neutral posture snout	The actor brings his nose close to or pushes it towards the nostrils of the recipient while being in a neutral posture.
Neutral posture face lick	The actor licks the nose, lips and mandibular region of the recipient while being in a neutral posture.
Pass under head	The actor passes from a lateral side close under the head of the recipient, usually in a somewhat crouching manner; often a short nose-chin contact with the recipient is evident.
Head under	The actor pushes with his head towards the ventro-lateral side of the recipient, occasionally lifting the recipient's back quarters from the ground with his head.
Fur sniff/licking	Self-explanatory
Paw/head on	The actor places a paw or its head on the back of the recipient.
Grin	Only clear facial expression shown by AWDs. The mouth corners remain retracted and the mouth may be slightly open so that the teeth become visible. The behaviour occurs mostly in combination with 'giggle' and has a friendly nature.
Giggle	A vocalisation characterised by a high tone level and a 'staccato' rhythm. The sound is made often in combination with the behaviour 'Grin'.
Greeting	The actor stands or walks in parallel with the recipient, tries to contact his muzzle, and performs a complex of behaviours including food-solicit or inspection behaviour, the facial expression 'grin' and the vocalisation 'giggle'.
Parallel walk or run	Two animals walking side by side in the same direction.
Regurgitation	Expulsion of undigested food from the mouth, pharynx, or oesophagus.
Rub on	The actor establishes intensive latero-lateral contact with the recipient. This may occur while both animals are in motion.
***Play behaviour***	
Play solicit*	The actor initiates a play interaction with the recipient by solicit behaviour such as nose pushing or tugging the recipients fur with an inhibited bite.
Fur bite*	The actor tugs the recipient fur by an inhibited bite.
Play fighting*	Playful non-competitive fighting in which attacker and defender exchanges roles and no winner or loser emerges. Interactions rarely include behaviours that can inflict injury.
Play chase*	The actor follows in a fast pursuit the recipient, who tries to escape by abruptly changing the direction. The roles of follow and escape may change.
Play wrestle*	This behaviour involves all play situations, in which the actor shows inhibited bite movements towards the recipient, while he keeps the recipient in constant eye contact.
Play sniff*	Two or more pack members are engaged in extensive sniffing at an object or some particle on the ground, while their heads are in direct contact.
Social play*	All other forms of play.

^a^Behaviours included in non-contact dominant behaviour

^b^Behaviours included in contact dominant behaviour

^c^Behaviours included in passive submission behaviour

^d^Behaviours included in active submission behaviour

*Behaviours not observed (severe aggressive behaviour, hoo call) or not included in analysis (play behaviour, food associated behaviour, behaviours that are a state e.g. close contact lying).

### Statistical analysis

All fGCM and fAM data was tested for normality using histograms and the Shapiro-Wilk test, and data were log_10_ transformed where not normally distributed. For EIA validation, differences in fGCM concentrations before and after the SIR-procedure were analysed with a paired sample t-test, while differences in fAM concentrations between male and female samples were analysed using an independent sample t-test. For the sample storage experiment, normalised relative fAM and fGCM levels were calculated by dividing every individual value across all time and temperature treatments by the mean hormone value (n = 3) at t = 0 h. Separate generalised linear models (glms) were run to analyse the levels of fGCM and fAM in samples over time (0–72 h and subsequently 0–24 h) and across the different temperature treatments (refrigeration vs. ambient temperature). These models were set up such that: y (fGCM or fAM) = sample (treated as a blocking factor including the 3 samples taken for each time point) + time (treated as linear). The pattern of the residuals for these models were examined and adjusted for within each model. For evaluating the effect of DAP *vs*. placebo treatment, normalised relative fGCM and fAM levels were calculated by dividing every individual value across all collection days and pheromone treatments by the mean pre-SIR hormone value (comprising the 4 sampling days pre-SIR; Fig 1) for each dog each season. By so doing, this controlled for potential individual animal differences in baseline fAM or fGCM concentrations, which could be due to a variety of reasons including the pack, an individual’s social status, the season, or female presence [39]. Normalised relative data were then grouped by intervention stage for each treatment according to (a) all pre-SIR-procedure samples (pre-SIR); (b) the first faecal sample after the SIR-procedure, reflecting hormone levels associated with the procedure (SIR); (c) the first following sample (SIR+1); and (d) all subsequent samples (SIR≥2; Fig 1). Differences in hormone levels between DAP *vs*. placebo treatment were then evaluated using an independent sample t-test. Differences between intervention stages were evaluated using one-way ANOVA and post-hoc Bonferroni.

Hourly rates of each behaviour type were calculated for each pack each day. Normalised relative hourly rates of behaviour were calculated by dividing every hourly rate per day across all observations days and pheromone treatments by the mean pre-SIR hourly rate (comprising the 3 observation days pre-SIR; Fig 1) for each pack each season. Day 1 (Fig 1) included the reintroduction of the pack and the first day of observation post-treatment. As we were unable to video record the reintroductions for the BRK pack, this pack was excluded from day 1 data. Since behavioural data (tested for normality as described above) were not normally distributed, differences in rates of behaviour between DAP *vs*. placebo treatment, as well as differences in rates of behaviour between Day 1 and mean pre-SIR baseline levels were then evaluated using a Mann-Whitney U test. Changes over time were evaluated using Friedman test combined with post-hoc Wilcoxon signed-rank test. Statistical analysis was performed with SPSS Statistics 23 (IBM SPSS Statistics 23, SPSS Inc., IBM, Armonk, New York, USA). *P* ≤ 0.05 was considered significant.

## Results

### EIA validation and steroid degradation experiment

**Fig 3:**
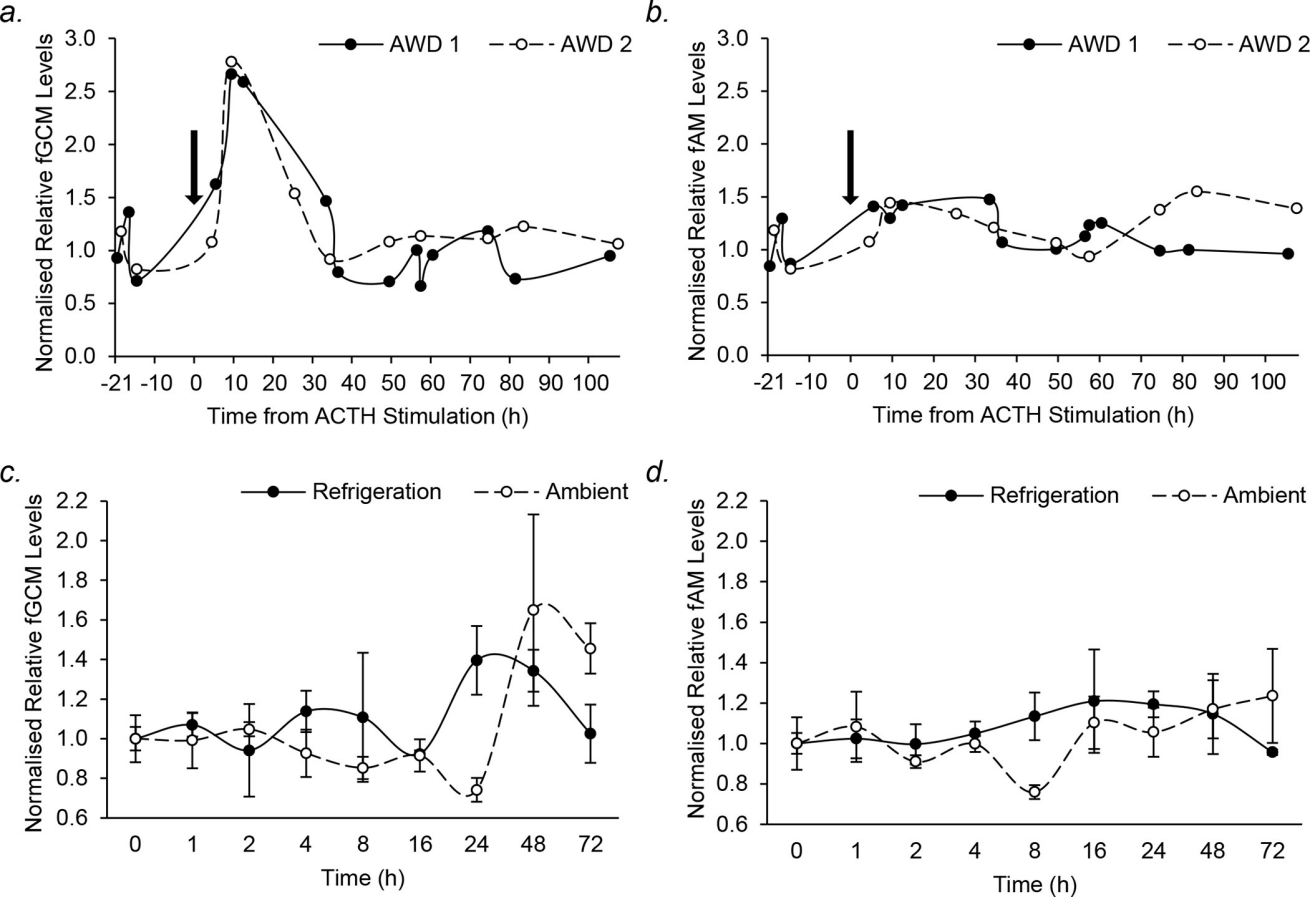
**Normalised relative (a) fGCM and (b) fAM profiles from n = 2 African wild dog females injected with ACTH (arrow); Mean (± SEM) normalised relative (c) fGCM and (d) fAM levels measured over 72 h after holding faecal samples at refrigerated (solid line) or ambient (dashed line) temperatures.** No significant differences between t = 0 h *vs*. t = 1, 2, 4, 8, 16, 24, 48, or 72 h in fGCM and fAM levels for either temperature treatment (*P* > 0.05).

ACTH injection resulted in detection of a 2.7-fold increase in fGCM levels by 8 h, which declined to baseline within 35 h. Cross-reactivity to fGCMs by the fAM assay was excluded since no rise in fAM levels were detected over the same period (Fig 3A and 3B). Moreover, mean fGCM concentrations after acute stress associated with the SIR procedure were significantly higher than pre-SIR baseline concentrations (paired sample t-test; SIR: 62.1 ± 8.6 ng/g DW; pre-SIR: 36.1 ± 2.7 ng/g DW; *t*_*9*_ = -2.527, *P* = 0.032). Furthermore, the fAM assay detected sex-specific differences in androgen concentrations with 10-fold higher fAM concentrations in male than female samples (independent t-test; male: 611.9 ± 41.1 ng/g DW; female: 62.9 ± 2.3 ng/g DW; *t*_*123*_ = 26.233, *P* < 0.001). Collectively these results provide experimental and biological validation that the cortisol-3-CMO and T-3-CMO assays are suitable for analyzing fGCM and fAM levels respectively in AWDs.

During the steroid degradation experiment, refrigeration temperature remained at 3–4°C except at 16 h and 72 h when it decreased to 1°C and 2°C respectively. External ambient summer temperatures ranged between 11°C at night to over 50°C (beyond the range of the thermometer) in direct sun during the day. Glm analyses found no significant effect of time on fGCM (*P* = 0.835) or fAM (*P* = 0.593) levels in samples stored at ambient temperatures over a 72 h period (Fig 3C and 3D). However, fAM levels differed significantly over time in refrigerated samples (*P* = 0.029), with an increased from 0 to 24 and 48 h, then a decrease again at 72 h. A similar pattern was observed in refrigerated fGCM samples over time, but this was not significant (*P* = 0.089). Since sampling in subsequent experiments occurred within a 24 h window, glm analyses confined to the 0 to 24 h period found no significant effect of time on fGCM or fAM levels in samples regardless of the type of storage temperature used (all *P* > 0.05).

### fGCM and fAM hormone levels

**Fig 4:**
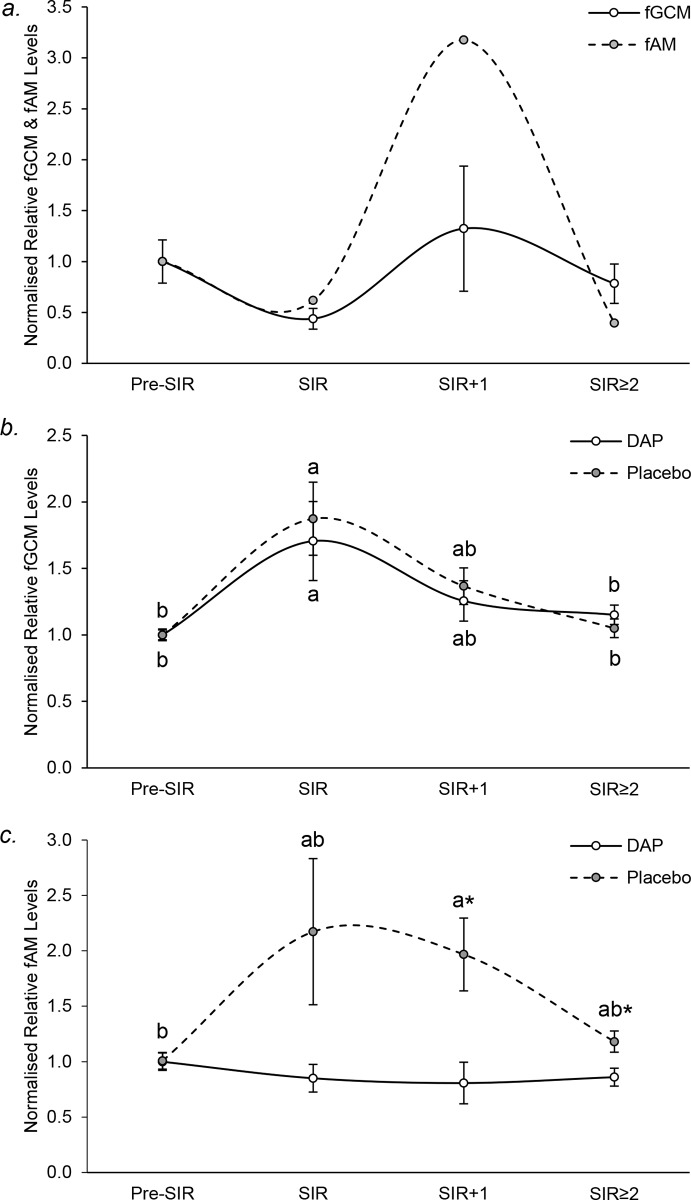
Mean (± SEM) normalised relative hormone levels before (pre-SIR) and after (SIR, SIR+1, SIR≥2) separation, immobilisation and reintroduction. (a) fGCM (n = 2 African wild dog females and 1 male; solid line) and fAM (n = 1 male; dashed line) in non-immobilised animals; (b) fGCM and (c) fAM in n = 11 DAP (solid line) or n = 12 placebo (dashed line) treated males; different letters indicate significant differences during the SIR procedure within each treatment; * significant differences between treatments at a particular intervention stage (*P* ≤ 0.05).

Three untreated animals that were not immobilised during the SIR procedure (BRK female in pre-breeding season; BRK male and OKC female in breeding season; Table 1), did not show a rise in fGCM levels (Fig 4A). Moreover, relative fGCM levels at SIR and SIR≥2 in these animals, were significantly lower than those in placebo treated animals (independent t-test; SIR, non-sedated animals: 0.4 ± 0.1; placebo: 1.9 ± 0.3, *t*_*13*_ = -4.251, *P* = 0.001; independent t-test; SIR≥2, non-sedated animals: 0.8 ± 0.2, placebo: 1.1 ± 0.1, *t*_*35*_ = -2.255, *P* = 0.030); indicating that immobilisation and not pack separation, isolation or pack reintroduction is responsible for the rise in fGCM seen after SIR in placebo treated animals (dashed line in Fig 4B). fAM levels in the single non-immobilised male, had a 3.2-fold increase at SIR+1 compared to baseline; indicating reintroduction of conspecifics is responsible for the rise in fAM (Fig 4A).

Among immobilised animals, a significant rise in fGCM of more than 1.5-fold occurred at SIR in both placebo (ANOVA; *F*_*3*,*92*_ = 12.306, *P* < 0.001) and DAP treated AWDs (ANOVA; *F*_*3*,*74*_ = 6.831, *P* < 0.001), with levels decreasing at SIR+1. There was no significant difference in fGCM between treatments (independent t-test; SIR: *t*_*21*_ = -0.422, *P* = 0.677; SIR+1: *t*_*16* =_ -0.516, *P* = 0.613; SIR≥2: *t*_*53*_ = 0.991, *P* = 0.326; Fig 4B). Compared to pre-SIR levels, placebo treated animals had between 1.2- to 2.2-fold higher fAM throughout the post-immobilisation period, with significantly higher levels observed at SIR+1 (ANOVA; *F*_*3*,*92*_ = 3.839, *P* = 0.012; Fig 4C). In contrast, this rise did not occur in DAP treated animals (ANOVA; *F*_*3*,*74*_ = 0.775, *P* = 0.512; Fig 4C), with fAM levels significantly lower than placebo treated animals at SIR+1 and SIR≥2 (independent t-test; SIR: *t*_*21*_ = -1.712, *P* = 0.097; SIR+1: *t*_*16*_ = -2.623, *P* = 0.018; SIR≥2: *t*_*53*_ = -2.464, *P* = 0.013; Fig 4C). The rise in fAM levels seen in placebo treated animals appeared to be independent of dominant status (SIR alpha males: 1.29 ± 0.86; SIR subordinate males: 2.61 ± 0.89).

### Behaviour

Mean daily observation time did not differ between placebo and DAP treated packs (Mann-Whitney U: *U* = 319, *N*_*1*_ *= N*_*2*_ = 27, *P* = 0.431; Table 1). For all packs combined, baseline rates of behaviour each hour pre-SIR were 2.2 ± 0.2 combined dominant behaviour; consisting of 0.5 ± 0.1 contact dominant, 0.8 ± 0.2 non-contact dominant and 0.9 ± 0.2 marking behaviours. Rates for combined submissive behaviour were 2.9 ± 0.5; consisting of 1.4 ± 0.3 active submissive and 1.4 ± 0.3 passive submissive behaviours. Affiliative behaviour was the most frequently observed at 4.2 ± 0.5 events per hour. Severe aggression was absent in all packs throughout the study period (including reintroduction), and ritualised aggression was low at 0.2 ± 0.1 events per hour.

**Fig 5:**
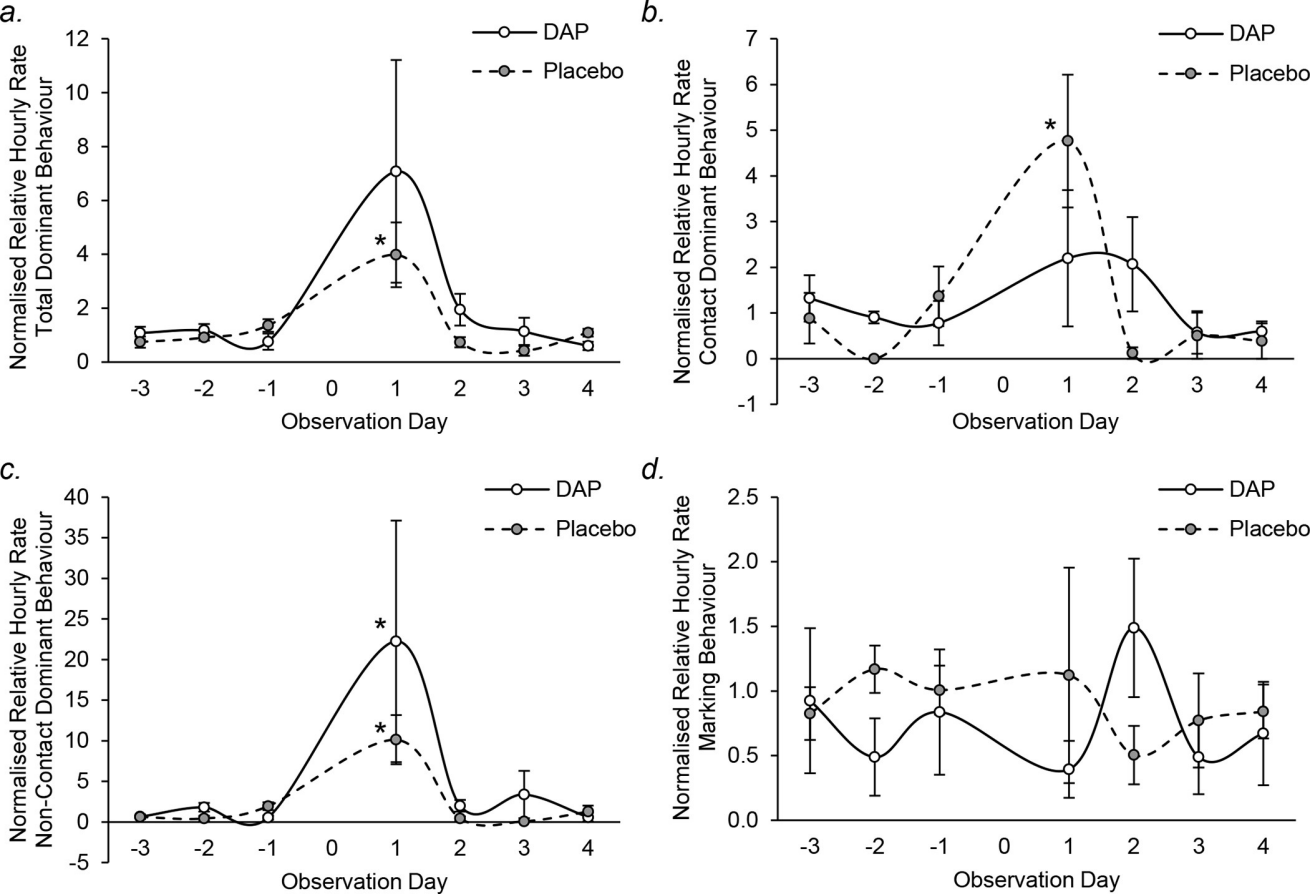
**Mean (± SEM) normalised relative hourly rates of (a) total, (b) contact and (c) non-contact dominance behaviour, and (d) marking in DAP (solid line) and placebo (dashed line) treated packs throughout the observation period**. * significantly different to respective baseline levels (*P* ≤ 0.05).

Relative hourly rates of dominant behaviour in DAP and placebo treated groups are shown in Fig 5. In placebo treated packs, total (Friedman test; Fig 5A, *χ*^*2*^ = 12.571, *P* = 0.050) and contact dominant behaviour (Friedman test; Fig 5B, *χ*^*2*^ = 13.304, *P* = 0.038) changed significantly over time, but the specific days could not be clarified by post hoc Wilcoxon signed-rank test. However, relative hourly rates of total, contact and non-contact dominant behaviour were significantly higher on the day of release (+1) than before intervention (Mann-Whitney U: total dominant behaviour: *U* = 0, *N*_*1*_ = 3, *N*_*2*_ = 12, *P* = 0.009, Fig 5A; contact dominant behaviour: *U* = 1, *N*_*1*_ = 3, *N*_*2*_ = 12, *P* = 0.010, Fig 5B; non-contact dominant behaviour: *U* = 0, *N*_*1*_ = 3, *N*_*2*_ = 12, *P* = 0.009, Fig 5C). In DAP treated packs, no dominant behaviour changed significantly over time (Fig 5), but non-contact dominant behaviour significantly increased on the day of release compared to baseline (Mann-Whitney U: *U* = 2, *N*_*1*_ = 3, *N*_*2*_ = 12, *P* = 0.021, Fig 5C). No changes could be seen in marking behaviour for either treatment group (Friedman test, Placebo: *χ*^*2*^ = 4.571, *P* = 0.600; DAP: *χ*^*2*^ = 8.595, *P* = 0.198; Fig 5D). Although no significant differences were observed in any dominant behaviours between placebo and DAP treated packs (Mann-Whitney U: total dominant behaviour day+1: *U* = 4, *N*_*1*_ = *N*_*2*_ = 3, *P* = 0.827, Fig 5A; contact dominant behaviour day+1: *U* = 2, *N*_*1*_ = *N*_*2*_ = 3, *P* = 0.275, Fig 5B; non-contact dominant behaviour day+1: *U* = 4, *N*_*1*_ = *N*_*2*_ = 3, *P* = 0.827, Fig 5C), placebo groups tended to exhibit 2.2-fold higher contact dominant behaviour (Fig 5B), while DAP groups tended to exhibit 2.2-fold higher non-contact dominant behaviour (Fig 5c).

**Fig 6:**
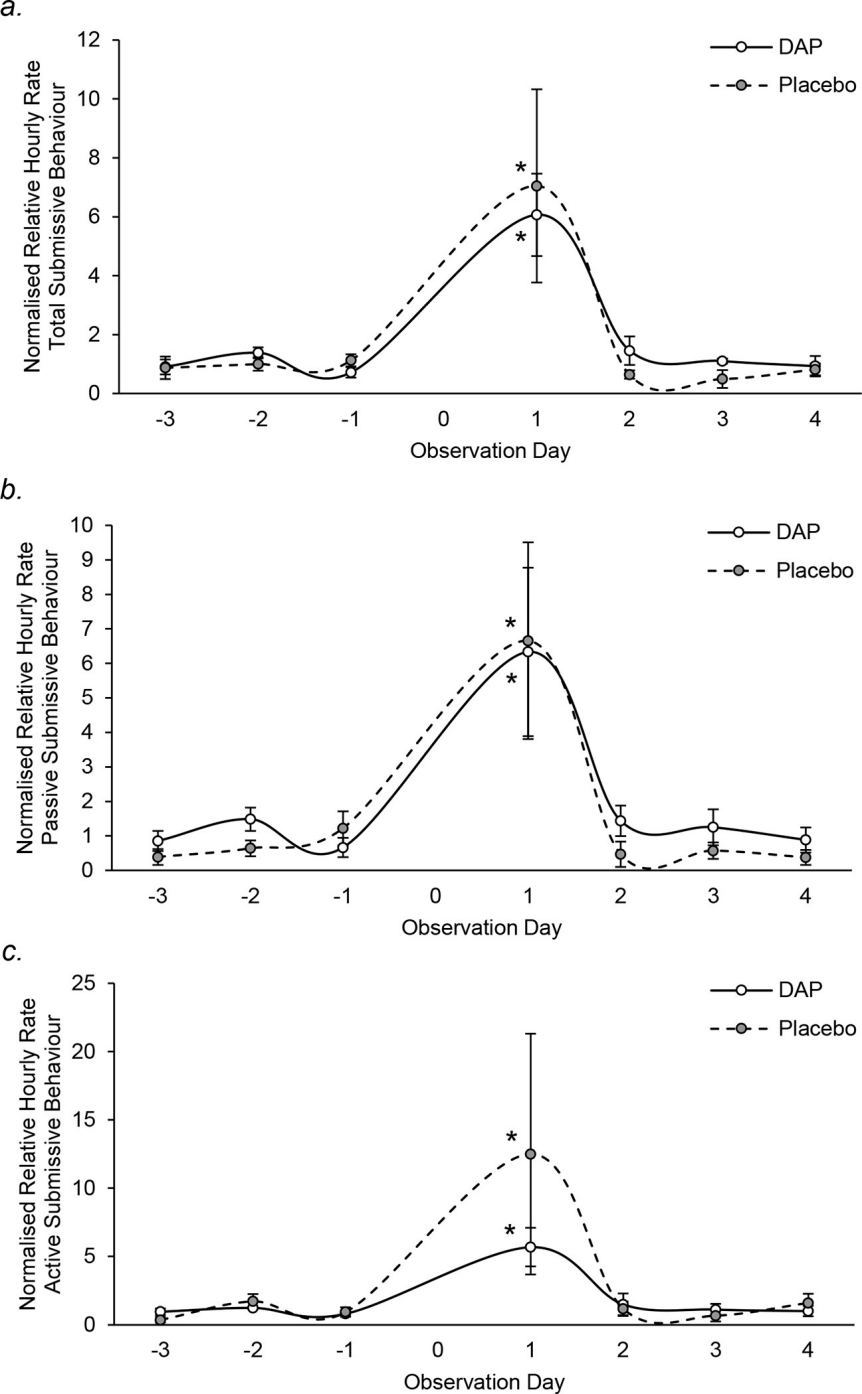
**Mean (± SEM) normalised relative hourly rates of (a) total, (b) passive and (c) active submissive behaviour in DAP (solid line) and placebo (dashed line) treated packs throughout the observation period.** * significantly different to respective baseline levels (*P* ≤ 0.05).

Relative hourly rates of total or passive submissive behaviour did not change significantly over time in either treatment group (Fig 6A and 6B). However, changes in active submission over time were near-significant for the placebo group (*χ*^*2*^ = 11.122, *P* = 0.085), but not DAP group (*χ*^*2*^ = 7.714, *P* = 0.260; Fig 6C). However, total, active and passive submission was significantly higher on the day of release than before intervention for both treatments (*P* ≤ 0.05; Fig 6). Although no significant differences were observed in any submissive behaviours between placebo and DAP treated packs (Mann-Whitney U: total submissive behaviour day+1: *U* = 4, *N*_*1*_ = *N*_*2*_ = 3, *P* = 0.827, Fig 6A; passive submissive behaviour day+1: *U* = 4, *N*_*1*_ = *N*_*2*_ = 3, *P* = 0.827, Fig 6B; active submissive behaviour day+1: *U* = 4, *N*_*1*_ = *N*_*2*_ = 3, *P* = 0.827, Fig 6C), placebo groups tended to exhibit 2.2-fold higher active submissive behaviour (Fig 6C).

**Fig 7:**
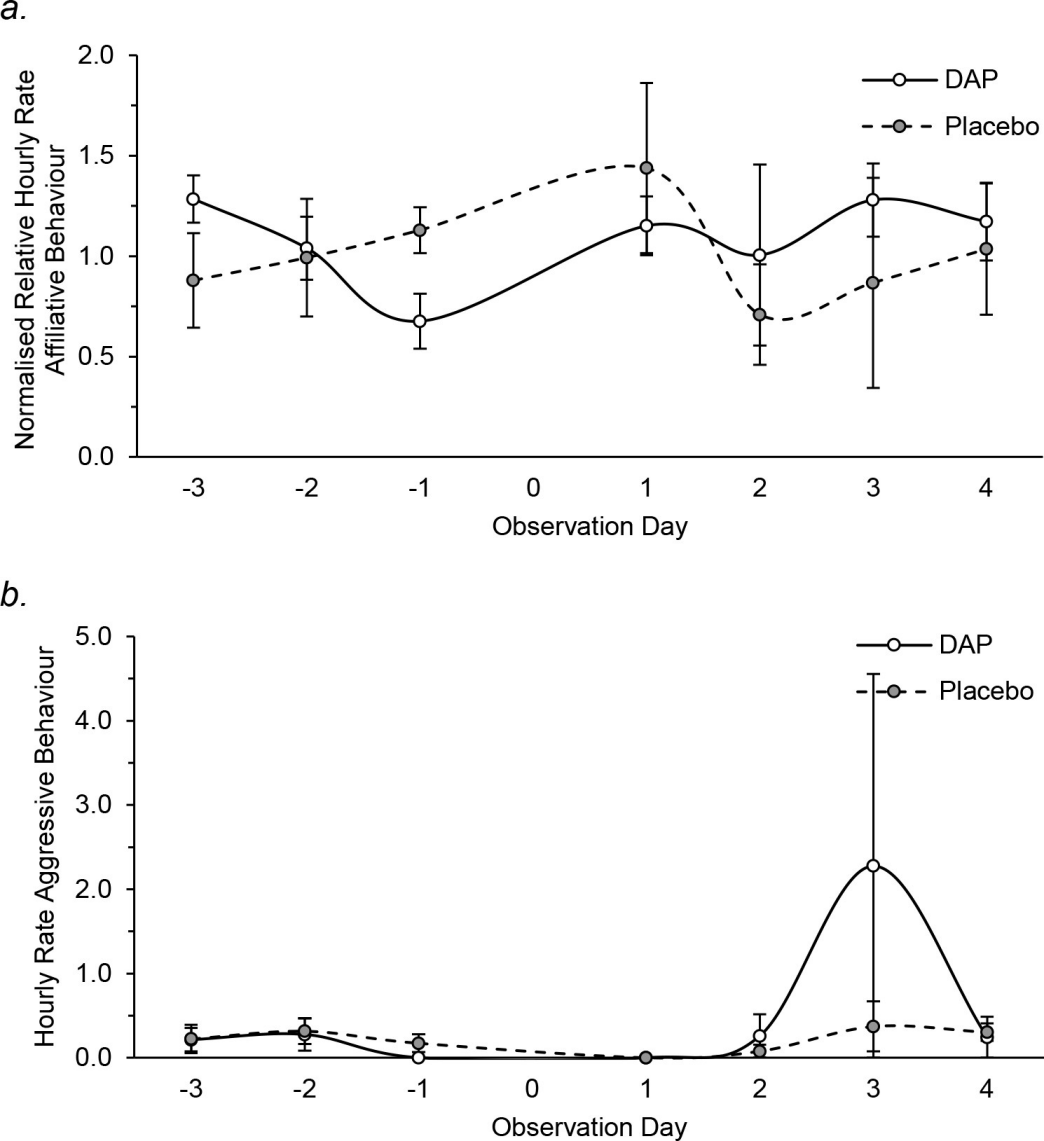
**Mean (± SEM) normalised relative hourly rates of (a) affiliative behaviour and hourly rates of (b) aggressive behaviour in DAP (solid line) and placebo (dashed line) treated packs throughout the observation period.** Not significantly different to respective baseline levels (*P* > 0.05).

With the exception of a spike in ritualised aggression observed on Day 3 in the ABQ pack (caused by a delayed restoration of normal hierarchy due to a transient shift), relative hourly rates of both affiliative and aggressive behaviour did not change significantly over time nor between placebo and DAP treated packs (Fig 7).

## Discussion

Dog appeasing pheromone offers tremendous potential for the management of behaviour in wild canids. Given its role in alleviating stress and anxiety-related behaviours in domestic dogs [26–32], DAP was presumed to act via the hypothalamic-pituitary-adrenal (HPA) axis to suppress cortisol production. Interestingly, our double-blinded placebo-controlled study demonstrates that DAP does not mitigate the faecal glucocorticoid surge associated with stressful interventions. In contrast, DAP prevents the rise in faecal androgens seen in placebo treated animals after separation, immobilisation and reintroduction procedures. Moreover, on the day of reintroduction, DAP treated packs tended to have lower rates of contact-dominance and active-submission behaviour, but higher rates of non-contact dominance behaviour. These results instead suggest that DAP could reduce hormones and behaviours leading to aggression in captive African wild dogs.

To our knowledge, this is the first time androgens have been measured during a pack reintroduction event in AWDs; with a significant increase in fAMs observed in placebo treated animals. Increased testosterone is associated with competitive interaction [46] and elevates the risk of agonistic interactions between individuals. Although not significant, placebo treated animals experiencing the testosterone surge, tended to have higher rates of contact dominant and active submission behaviour. In addition, total and contact dominant behaviour was significantly higher compared to baseline levels in placebo but not in DAP treated packs. The increase in faecal androgen was seen in most placebo treated animals seemingly irrespective of their dominance status; with dominant behaviours exerted by more dominant males and submissive behaviours exerted by subdominants. Recent studies in humans show, however, that the relationship between testosterone and agonistic behaviour is not always straightforward. Testosterone can also enhance prosocial behaviours if these promote increasing social status [47]. In addition, increased testosterone enhances dominant behaviour in men with high social status but causes strategic submission in socially lower-ranked individuals [48]. A similar effect in the placebo-treated AWD packs in this study could explain the tendency for subdominants to show higher active submission, while also showing higher testosterone levels.

Given that faecal samples collected at SIR reflect fAM concentrations accumulated across the entire separation, immobilisation and reintroduction procedure, it could be argued that the higher levels in placebo treated animals might not necessarily be caused by the reintroduction itself. In our study, treatment was only applied at the end of anaesthesia, to reduce stress related to the reintroduction. DAP therefore, could not suppress any putative increase in fAMs induced by capture or anaesthesia. However, studies in other species show that testosterone actually decreases during capture [49]. Moreover, we saw a similar rise in fAMs in the AWD male that was not sedated; suggesting that the increase in fAMs in the placebo group is caused by the reintroduction event.

No differences in fGCM levels could be seen between DAP and placebo treated animals, with both having a significant peak in response to the SIR-procedure. Faecal samples collected at SIR reflect stress hormones accumulated across the entire separation, immobilisation and reintroduction procedure. As such, due to the time of treatment, DAP could not suppress any cortisol produced by stressful events associated with capture and immobilisation. Serial blood samples drawn from AWDs after chemical immobilisation show a rise in cortisol concentrations for up to 40 min post-darting [10]. Moreover, despite being subjected to similar levels of stress caused by pack separation and reintroduction, the 3 non-sedated animals in our study failed to show a similar fGCM peak; suggesting that the increase in fGCM in both placebo and DAP treated animals is caused by the immobilisation event prior to the application of DAP. This explains the lack of difference between the 2 treatment groups. However, given the mechanism of pheromone action, it is unlikely that DAP would be effective in mitigating the cortisol rise in an unconscious animal.

Our study has also demonstrated that steroids such as fGCM and fAM appear to be highly stable in faeces held in fluctuating ambient temperatures up to 50°C for up to 72 h, while storage of faecal samples on ice (~4°C) appears to be stable for up to 24 h. This has important implications for faecal sample collection and hormone analysis of AWDs in the field.

Most domestic dog studies use behaviour to evaluate the effectiveness of DAP; which has been shown to elicit positive behaviour in adult dogs subjected to a variety of fear- and stress-inducing situations [26–32]. However, such studies have not examined the physiological/hormonal response to the pheromone, so its exact underlying mechanism of action is still unknown. The vomeronasal organ transduces pheromonal signals to the amygdala and hypothalamus [22], but the neurological pathways involved in signal transduction are not understood. Prolactin decreases in placebo, but less in DAP treated domestic dogs after surgery, but no effect was observed in any other parameter, including salivary cortisol [33].

As little is known about the underlying mechanism of action of DAP, it is unclear how it suppressed fAMs after intervention in our study. It is possible that DAP may act directly on the hypothalamic-pituitary-gonadal (HPG) axis. However, the effect could be mediated by elevated prolactin concentrations in DAP treated animals [33], known to decrease testosterone secretion by inhibiting GnRH neurons [50]. Moreover, prolactin increases in domestic dogs during positive, affiliative interactions with humans [51]. Thus, it is possible that DAP may upregulate the prolactin pathway, thereby triggering positive behavioural experiences, while simultaneously suppressing testosterone-mediated aggressive or dominant behaviours. Further studies are necessary in domestic dogs to determine these mechanisms.

A major limitation when working with any endangered species with such complex behaviour like the AWD, is access to sufficiently large numbers of packs/animals to evaluate behaviour. In our study, we had to exclude behavioural analysis at the time of reintroduction in one pack for both the DAP and placebo treatment; leaving only 3 packs in each group to evaluate the behavioural effect of DAP on the day of reintroduction. There were however, significant changes on the day of release compared to baseline levels, and trends towards different behaviours between DAP and placebo treated packs were evident. We believe that increasing the sample size will reveal significant differences in behaviour between the 2 treatments.

Although appeasing pheromones are thought to be species-specific, they have the same core chemical composition in different species. Three fatty acids (oleic acid, palmitic acid, and linoleic acid) appear in the same ratio across all species. Other fatty acids then follow in a species-specific ratio that are, for the domestic dog, identified as myristic acid, lauric acid, pentadecanoic acid, and stearic acid [22]. Given the similarity of these core fatty acids, it is possible that a highly concentrated species-specific pheromone could have an effect on a related species [9, 52]; which has been confirmed in this study. However, it is possible an even more prominent effect on behaviour could be seen in response to an AWD-specific appeasing pheromone, which is yet to be developed. Thus, future parallel studies should focus on isolation and testing of such wild dog-specific pheromones.

In our study, little to no aggression occurred during reintroduction events involving both DAP or placebo treated packs. Aggression after temporary pack separation is quite common in captive AWDs [4, 6–9], and is the reason why many zoological institutions are reluctant to immobilise animals in order to participate in conservation research or even in annual health check-ups [8]. However, this and previous studies [8], show that when separation and reintroduction is well managed, it can be performed without major issues. We advocate that DAP can play an important role to reduce these risks further. Reintroductions in this study were typically characterised by a peak in dominant and submissive behaviour at reintroduction. Affiliative behaviour remained close to baseline levels, and aggression was almost completely absent during the study period. One of the factors thought to improve our success during reintroduction was the isolation of all pack members during intervention, thereby avoiding the formation of different social bonds between individuals. In contrast, 2 subdominant males were reintroduced to each other in the ABQ pack during the breeding season without the dominant male, who was released the next day. This caused a hierarchy shift of the dominant male to the lowest rank without any sign of aggression. Three days after reintroduction, the original hierarchy was re-established, resulting in an episode of ritualised aggression from the alpha and beta male toward the original gamma male. To avoid similar situations, we advise the separation of all AWDs where group size and infrastructure permits it, combined with the simultaneous reintroduction of all members of the pack.

In conclusion, in our study, DAP prevented the faecal androgen but not faecal cortisol surge in AWDs caused by stressful interventions. This was associated with a trend for lower rates of contact dominance and active submission. These effects could decrease the risk of agonistic interactions and suggest that DAP may be a useful tool to help manage new pack formations and temporary pack separation. However, more research is needed using increased sample size or AWD-specific appeasing pheromones to conclusively elucidate the behavioural effects of DAP and its underlying mechanism of action.

## References

[1] Creel S, Creel NM, editors. The African Wild Dog Behavior, Ecology, and Conservation. Princeton: University Press; 2002.

[2] Van den Berghe F, Paris DB, Van Soom A, Rijsselaere T, Van der Weyde L, Bertschinger HJ, et al Reproduction in the endangered African wild dog: basic physiology, reproductive suppression and possible benefits of artificial insemination. Anim Reprod Sci. 2012;133(1–2):1–9. 10.1016/j.anireprosci.2012.06.003 22748701

[3] Davies-Mostert HT, Mills MGL, Macdonald DW. A Critical Assessment of South Africa's Managed Metapopulation Recovery Strategy for African Wild Dogs and its value as a template for large carnivore conservation elsewhere In: Hayward MW, Somers MJ, editors. Reintroduction of Top-Order Predators. Londen: Wiley-Blackwell; 2009.

[4] Scheepers JL, Venzke KAE. Attempts to reintroduce African wild dogs *Lycaon pictus* into Etosha National Park, Namibia. S Afr J Wildl Res. 1995;25:138–40.

[5] Woodroffe R, Ginsberg J. Conserving the African wild dog *Lycaon pictus*. II. Is there a role for reintroduction? Oryx. 1999;33:143–51.

[6] Foster B. Challenges faced while trying to promote pack unity in 3.1 painted dogs at the Oregon zoo. African painted dog conference; April 29-May 2, 2014; Chicago, USA. p. 27.

[7] Quick M. African painted dog SSP husbandry review. African painted dog conference; April 29-May 2, 2014; Chicago, USA. p. 21.

[8] Johnston SD, Ward D, Lemon J, Gunn I, MacCallum CA, Keeley T, et al Studies of male reproduction in captive African wild dogs (*Lycaon pictus*). Anim Reprod Sci. 2007;100(3–4):338–55. 10.1016/j.anireprosci.2006.08.017 16987622

[9] Vlamings BHAC. Dog appeasing pheromone: A useful tool to minimize stress and aggression of African wild dogs (Lycaon pictus)?: Graduate School of Life Sciences, University of Utrecht, the Netherlands; 2011 https://ibream.org/wp-content/uploads/2018/04/Vlamings-2011-UU-MSc-thesis-DAP-to-minimize-stress-aggression-in-AWD....pdf.

[10] de Villiers MS, van Jaarsveld AS, Meltzer DGA, Richardson PRK. Social dynamics and the cortisol response to immobilization stress of the African wild dog, *Lycaon pictus*. Horm Behav. 1997;31:3–14. 10.1006/hbeh.1997.1314 9109594

[11] de Villiers MS, Meltzer DGA, Van Heerden J, Mills MGL, Richardson PRK, Van Jaarsveld AS. Handling-induced stress and mortalities in African wild dogs (*Lycaon pictus*). Proc R Soc Lond [Biol]. 1995;262(1364):215–20. 10.1098/rspb.1995.0198 8524913

[12] Comizzoli P, Crosier AE, Songsasen N, Gunther MS, Howard JG, Wildt DE. Advances in reproductive science for wild carnivore conservation. Reprod Domest Anim. 2009;44:47–52. 10.1111/j.1439-0531.2009.01373.x 19754535

[13] Creel S, Creel NM, Monfort SL. Radiocollaring and stress hormones in African wild dogs. Conserv Biol. 1997;11(2):544–8.

[14] Beerda B, Schilder MBH, Van Hooff JARAM, De Vries HW, Mol JA. Chronic stress in dogs subjected to social and spatial restriction. I. Behavioral responses. Physiology & Behavior. 1999;66(2):233–42. 10336149 10.1016/s0031-9384(98)00289-3

[15] Rosado B, Garcia-Belenguer S, Leon M, Chacon G, Villegas A, Palacio J. Blood concentrations of serotonin, cortisol and dehydroepiandrosterone in aggressive dogs. Appl Anim Behav Sci. 2010;123(3–4):124–30. 10.1111/j.1365-2885.2010.01254.x 21198677

[16] Creel S, Creel NM, Mills MGL, Monfort SL. Rank and reproduction in cooperatively breeding African wild dogs: Behavioral and endocrine correlates. Behav Ecol. 1997;8(3):298–306.

[17] Mehta PH, Beer J. Neural mechanisms of the testosterone-aggression relation: The role of orbitofrontal cortex. J Cogn Neurosci. 2010;22(10):2357–68. 10.1162/jocn.2009.21389 19925198

[18] Wacker DW, Khalaj S, Jones LJ, Champion TL, Davis JE, Meddle SL, et al Dehydroepiandrosterone heightens aggression and increases androgen receptor and aromatase mRNA expression in the brain of a male songbird. J Neuroendocrinol. 2016;28(12):9. 10.1111/jne.12443 PMC5333462 27805753

[19] Maarschalkerweerd RJ, Endenburg N, Kirpensteijn J, Knol BW. Influence of orchiectomy on canine behaviour. Vet Rec. 1997;140(24):617–9. 9228691 10.1136/vr.140.24.617

[20] delBarco-Trillo J, Greene LK, Braga Goncalves I, Fenkes M, Wisse JH, Drewe JA, et al Beyond aggression: Androgen-receptor blockade modulates social interaction in wild meerkats. Horm Behav. 2016;78:95–106. 10.1016/j.yhbeh.2015.11.001 26545817

[21] Monfort SL, Wasser SK, Mashburn KL, Burke M, Brewer BA, Creel SR. Steroid metabolism and validation of noninvasive endocrine monitoring in the African wild dog (*Lycaon pictus*). Zoo Biol. 1997;16(6):533–48.

[22] Pageat P, Gaultier E. Current research in canine and feline pheromones. Vet Clin North Am Small Anim Pract. 2003;33(2):187–211. 12701508 10.1016/s0195-5616(02)00128-6

[23] Yonezawa T, Koori M, Kikusui T, Mori Y. Appeasing pheromone inhibits cortisol augmentation and agonistic behaviors during social stress in adult miniature pigs. Zool Sci. 2009;26(11):739–44. 10.2108/zsj.26.739 19877832

[24] Mengoli M, Pageat P, Lafont-Lecuelle C, Monneret P, Giacalone A, Sighieri C, et al Influence of emotional balance during a learning and recall test in horses (*Equus caballus*). Behav processes. 2014;106:141–50. 10.1016/j.beproc.2014.05.004 24875282

[25] Pereira JS, Fragoso S, Beck A, Lavigne S, Varejao AS, da Graca Pereira G. Improving the feline veterinary consultation: the usefulness of Feliway spray in reducing cats' stress. J Feline Med Surg. 2016;18(12):959–64. 10.1177/1098612X15599420 26282847 PMC11112237

[26] Sheppard G, Mills DS. Evaluation of dog-appeasing pheromone as a potential treatment for dogs fearful of fireworks. Vet Rec. 2003;152(14):432–6. 12708592 10.1136/vr.152.14.432

[27] Levine ED, Ramos D, Mills DS. A prospective study of two self-help CD based desensitization and counter-conditioning programmes with the use of Dog Appeasing Pheromone for the treatment of firework fears in dogs (*Canis familiaris*). Appl Anim Behav Sci. 2007;105(4):311–29.

[28] Landsberg GM, Beck A, Lopez A, Deniaud M, Araujo JA, Milgram NW. Dog-appeasing pheromone collars reduce sound-induced fear and anxiety in beagle dogs: a placebo-controlled study. Vet Rec. 2015;177(10):260 10.1136/vr.103172 26311736 PMC4602264

[29] Gandia Estelles M, Mills DS. Signs of travel-related problems in dogs and their response to treatment with dog-appeasing pheromone. Vet Rec. 2006;159(5):143–8. 16877680 10.1136/vr.159.5.143

[30] Tod E, Brander D, Waran N. Efficacy of dog appeasing pheromone in reducing stress and fear related behaviour in shelter dogs. Appl Anim Behav Sci. 2005;93(3–4):295–308.

[31] Kim YM, Lee JK, Abd El-aty AM, Hwang SH, Lee JH, Lee SM. Efficacy of dog-appeasing pheromone (DAP) for ameliorating separation-related behavioral signs in hospitalized dogs. Can Vet J. 2010;51(4):380–4. 20592826 PMC2839826

[32] Mills DS, Ramos D, Estelles MG, Hargrave C. A triple blind placebo-controlled investigation into the assessment of the effect of Dog Appeasing Pheromone (DAP) on anxiety related behaviour of problem dogs in the veterinary clinic. Appl Anim Behav Sci. 2006;98(1–2):114–26.

[33] Siracusa C, Manteca X, Cuenca R, del Mar Alcala M, Alba A, Lavin S, et al Effect of a synthetic appeasing pheromone on behavioral, neuroendocrine, immune, and acute-phase perioperative stress responses in dogs. J Am Vet Med Assoc. 2010;237(6):673–81. 10.2460/javma.237.6.673 20839989

[34] Gaultier E, Bonnafous L, Vienet-Legue D, Falewee C, Bougrat L, Lafont-Lecuelle C, et al Efficacy of dog-appeasing pheromone in reducing stress associated with social isolation in newly adopted puppies. Vet Rec. 2008;163(3):73–80. 18641375 10.1136/vr.163.3.73

[35] Taylor K, Mills DS. A placebo-controlled study to investigate the effect of Dog Appeasing Pheromone and other environmental and management factors on the reports of disturbance and house soiling during the night in recently adopted puppies (*Canis familiaris*). Appl Anim Behav Sci. 2007;105(4):358–68.

[36] Gaultier E, Bonnafous L, Vienet-Lague D, Falewee C, Bougrat L, Lafont-Lecuelle C, et al Efficacy of dog-appeasing pheromone in reducing behaviours associated with fear of unfamiliar people and new surroundings in newly adopted puppies. Vet Rec. 2009;164(23):708–14. 19502626 10.1136/vr.164.23.708

[37] Denenberg S, Landsberg GM. Effects of dog-appeasing pheromones on anxiety and fear in puppies during training and on long-term socialization. J Am Vet Med Assoc. 2008;233(12):1874–82. 10.2460/javma.233.12.1874 19072600

[38] Gaultier E, Bonnafous L, Bougrat L, Lafont C, Pageat P. Comparison of the efficacy of a synthetic dog-appeasing pheromone with clomipramine for the treatment of separation-related disorders in dogs. Vet Rec. 2005;156(17):533–8. 15849342 10.1136/vr.156.17.533

[39] Van den Berghe F, Paris MCJ, Sarnyai Z, Briggs MB, Millar RP, Ganswindt A, Paris DBBP. Social rank does not affect sperm quality in male African wild dogs (*Lycaon pictus*). Reprod Fert Dev. 2019; 10.1071/RD18205. 30694739

[40] Van den Berghe F, Paris MCJ, Briggs MB, Farstad WK, Paris DBBP. A two-step dilution tris-egg yolk extender containing Equex STM significantly improves sperm cryopreservation in the African wild dog (*Lycaon pictus*). Cryobiol. 2018;80:18–25. 10.1016/j.cryobiol.2017.12.095 29287895

[41] Van der Weyde LK, Martin GB, Blackberry MA, Gruen V, Harland A, Paris MC. Reproductive hormonal patterns in pregnant, pseudopregnant and acyclic captive African wild dogs (*Lycaon pictus*). Anim Reprod Sci. 2015;156:75–82. 10.1016/j.anireprosci.2015.03.003 25818522

[42] van der Goot AC, Martin GB, Millar RP, Paris MC, Ganswindt A. Profiling patterns of fecal 20-oxopregnane concentrations during ovarian cycles in free-ranging southern white rhinoceros (*Ceratotherium simum simum*). Anim Reprod Sci. 2015;161:89–95. 10.1016/j.anireprosci.2015.08.009 26372226

[43] Palme R, Mostl E. Measurement of cortisol metabolites in faeces of sheep as a parameter of cortisol concentration in blood. Z Saugetierkd-Int J Mamm Biol. 1997;62:192–7.

[44] Palme R, Mostl E. Biotin-Streptavidin Enzyme Immunoassay For The Determination Of Oestrogen And Androgens In Boar Faeces In: Görög ES, editor. Advances of steroid analysis ‘93; Akadémiai Kiadó, Budapest 1994 p. 111–7.

[45] Ganswindt A, Heistermann M, Borragan S, Hodges JK. Assessment of testicular endocrine function in captive African elephants by measurement of urinary and fecal androgens. Zoo Biol. 2002;21(1):27–36.

[46] Zilioli S, Bird BM. Functional significance of men’s testosterone reactivity to social stimuli. Front Neuroendocrinol. 2017;47:1–18. 10.1016/j.yfrne.2017.06.002 28676436

[47] Dreher JC, Dunne S, Pazderska A, Frodl T, Nolan JJ, O'Doherty JP. Testosterone causes both prosocial and antisocial status-enhancing behaviors in human males. Proc Natl Acad Sci U S A. 2016;113(41):11633–8. 10.1073/pnas.1608085113 27671627 PMC5068300

[48] Inoue Y, Takahashi T, Burriss RP, Arai S, Hasegawa T, Yamagishi T, et al Testosterone promotes either dominance or submissiveness in the Ultimatum Game depending on players' social rank. Sci Rep. 2017;7(1):5335 10.1038/s41598-017-05603-7 28706184 PMC5509644

[49] Lincoln G. Plasma testosterone profiles in male macropodid marsupials. J Endocrinol. 1978;77(3):347–51. 351124 10.1677/joe.0.0770347

[50] Gill-Sharma MK. Prolactin and Male Fertility: The long and short feedback regulation. Int J Endocrinol. 2009;2009:13. 10.1155/2009/687259 PMC2778443 20011060

[51] Odendaal JSJ, Meintjes RA. Neurophysiological correlates of affiliative behaviour between humans and dogs. Vet J. 2003;165(3):296–301. 12672376 10.1016/s1090-0233(02)00237-x

[52] Gaultier E, Falewee C, Bougrat L, Pageat P. The introduction of a female tiger (*Panthera tigris*) in a pre-established group of two neutered males: A case study Mills D, Levine E, Landsberg G, Horwitz D, Duxbury M, Mertens P, et al, editors. W Lafayette: Purdue Univ Press; 2005 1–5 p.

